# Comparative study of pulmonary functions test among different substances abusers

**DOI:** 10.1186/s12890-023-02760-6

**Published:** 2023-11-20

**Authors:** Hana Salah Musa Mohamed, Ibrahim Abdelrhim Ali

**Affiliations:** Department of Physiology, Faculty of Medicine, The National Ribat University, Khartoum, Sudan

**Keywords:** Pulmonary function tests, Cannabis, Heroin, Methamphetamine, Alcohol

## Abstract

**Background:**

Substance use is a problem that affects people all over the world and is prevalent in different age groups. The lungs in particular, with their unique exposure to the environment and the bloodstream, are vulnerable to damage from substance use and can affect lung function. Efforts have generally focused on cigarettes, while there is little research on different substances of use. The study aimed to detect changes in pulmonary function tests in different substance users.

**Methods:**

An analytical cross-sectional study was carried out among different substance users at the Abdalaal Elidridi Psychiatric Hospital. A total of 60 adults were included: 16 cannabis users, 16 heroin users, 16 methamphetamine users, and 12 alcohol users. Participants used only one substance. Height and weight were measured, and BMI was calculated. For each participant, pulmonary function tests (PFTs) including forced vital capacity (FVC), forced expiratory volume in one second (FEV1), FEV1/FVC ratio and peak expiratory flow rate (PEFR) were measured using an electronic spirometer, and the results were compared with normal reference values for Sudanese subjects matched for age, sex, and height.

**Results:**

A high prevalence of lung function abnormalities (100.0%) is seen in all abusers. Obstructive lung disease is found in 80.0% of patients, and restrictive lung disease is found in 20.0% of cases. The measured FEV1/FVC ratio in different substance groups was lower than the expected normal values of (88.2 ± 2.9%). The mean FEV1/FVC ratio of alcohol was (83.6 ± 7.6%), for heroin, it was (77.3 ± 15.8%), for methamphetamine, it was (77.7 ± 17.8%) and for cannabis, it was (71.03 ± 11.3%), the latter was significantly lower than the other two groups (P < 0.001). Duration of substance use was inversely correlated with the FEV1/FVC ratio (r = -0.378, P 0.001), indicating that a longer duration of substance use correlates with lower FEV1/FVC ratios.

**Conclusions:**

Obstructive lung abnormalities are frequent in substance abusers. All types of substances have a major deleterious effect on PFTs and harm the respiratory system. More action should be taken to address the effects of substances on the lungs. Awareness, early detection, and intervention are essential.

## Introduction

Substance use is a problem that affects people all over the world and is prevalent in different age groups. This is a problematic pattern of substance use that causes impairment or distress. Perhaps what we ingest, smoke, or otherwise put into our bodies can affect our health. Drugs of all kinds—prescription or street drugs—and other illegal substances can cause serious complications [1]. The lungs, in particular, are uniquely exposed to the environment and the bloodstream, making them susceptible to damage from drug use. This damage to the lungs can result from both systemic and inhalational exposure to various illicit drugs of abuse [2].

The pulmonary function tests (PFTs), including forced vital capacity (FVC), forced expiratory volume in 1 s (FEV1), forced expiratory flow (FEF), and FEV1/FVC ratio, are essential for the diagnosis of obstructive and restrictive respiratory diseases [3].

A descriptive-analytical study was carried out among drug users in Indonesia and showed a correlation between decreased FEV1/FVC and the duration of cannabis inhalation, methamphetamine inhalation, injected heroin, age, smoking duration, and cigarette consumption [4].

Regarding different types of substances, starting with heroin, Drummond et al. found in their study that 15.5% had airway obstruction, and heroin use was one of the risk factors for developing chronic obstructive pulmonary disease (COPD) [5]. The prevalence of COPD among habitual heroin smokers is much higher than among regular tobacco smokers in the same age group, and the average age of heroin smokers is also much lower than that of tobacco smokers at the time of COPD diagnosis [6].

In Amsterdam, Buster et al. reported on the difference between FEV1 and predicted values and found that heroin smokers had an FEV1 lower than the predicted FEV1 [7].

In several studies of heroin users, results show the significant respiratory impairment associated with heroin smoking and a worryingly accelerated rate of decline over time [8].

Cannabis smoking was associated with impairment of large airway function, resulting in airflow obstruction and hyperinflation. An association was found between cannabis smoking and a reduced FEV1/FVC ratio and specific airway conductance. For measures of airflow obstruction, one cannabis joint had a similar effect to 2.5–5 tobacco cigarettes [9].

In an early study by Tilles et al., 15 regular marijuana smokers were recruited to undergo spirometry assessments. In this case, both FEV1 and FVC were increased in marijuana smokers compared to non-smokers [10].

There is limited data on the relationship between lung function and the history of amphetamine use. Several cases have been reported of pan lobular emphysema in methamphetamine injectors [11]. Methamphetamine injection has been associated with pulmonary hypertension caused by contaminant embolization of the pulmonary vascular bed and can form foreign body granulomas [12]. Animal studies showed that inhalation of methamphetamine increased airway resistance, accompanied by a reduction in serotonin [13]. Children living near methamphetamine production areas showed transient asthma symptoms. [14].

Alcohol is known to adversely affect the lungs, with most observations focusing on the impairment of pulmonary host defenses [15] and the increased susceptibility of alcoholics to infection [16].

A meta-analysis using data from 15,294 study participants showed that the mean percent increase in FEV1 and FVC is higher in all categories of alcohol consumption compared to the reference group of ‘’lifetime non-drinkers’’, with the largest increase seen in subjects consuming ‘’5–14’’ or ‘’15–30’’, or 31–90’’ or ‘’more than 90’’ drinks per month (approximately 4.3% and 5% higher, respectively) and the smallest increase seen in subjects consuming ‘’less than 5’’ drinks per month. The mean FEV1/FVC is lower in all categories of alcohol consumption compared to the reference group of ‘’lifetime non-drinkers’’, with the largest decrease seen in subjects consuming ‘’more than 90’’ drinks per month [17].

Most of the previous studies looked at just one substance and did not compare the effects of different substances in the same study. Other substances, such as methamphetamine, were mainly covered by animal studies. The effects of these substances in relation to the duration of use have not been addressed. Observations made by Sudanese psychiatrists [18] sound the alarm of a dramatic increase in the number of drug abusers in Sudan (especially methamphetamine and cannabis [19]), making it even more the responsibility of researchers and medical teams to know the health dimensions of these dangerous substances in order to make appropriate medical decisions. This study aimed to detect changes in pulmonary function test among different substance users.

## Methods

An analytical cross-sectional study was conducted among different groups of drug users at the Abdalaal Elidridi Psychiatric Governmental Hospital which is affiliated to Ministry of Interior and the National Ribat University. This hospital located in Bahri town at Khartoum state and consists of 3 divisions: Mental department which consist of males Sect. (40 beds) and females Sect. (20 beds), Intensive care department for dangerous cases with special isolation (seclusion) rooms, and Psychiatric rehabilitations department. Each division or section is supervised by 2–4 psychiatric senior consultants or specialists, 3–6 psychiatric residents, 5 medical practitioners, 7–10 professional nurses, 2 psychologists, 2 social workers.

### Study population

A total of 60 participants aged 18–55 years were included: 16 cannabis users, 16 heroin users, 16 methamphetamine users, and 12 alcohol users. 55 males and only 5 females were included. These groups were selected using total coverage sampling during the study period of October 2022–December 2022. The sample size formula *n* = N * [Z2 * p * (1-p)/e2] / [N − 1 + (Z2 * p * (1-p)/e2] is required to know the adequate or correct proportion of the population, together with the confidence level and the margin of error.

### Inclusion criteria

Participants used only one substance.

### Exclusion criteria

Subjects with acute or chronic lung disease, respiratory infections, asthma, tuberculosis, and rheumatoid arthritis were excluded. In addition, all participants with multiple substance use disorders were excluded. COVID-19 infection was also excluded by viral isolation screening (from nasal or throat specimens and/or positive serological tests for IgG and IgM antibodies to COVID-19) as part of the routine work and pre-admission protocol of the Abdalaal Elidridi Psychiatric Hospital. Subjects who were febrile or had an infection within the previous month were also excluded. All participants were under medical supervision and had undergone a complete medical examination and laboratory investigations prior to enrollment in the study.

### Data collection

An interview questionnaire was completed to obtain relevant data, including age, sex, detailed medical history, chronic diseases, social habits, type of substance used, duration of substance use, and route of administration. Height was measured using a standardized scale, and PFTs (FEV1, FVC, FEV1/FVC ratio, and PEFR) were measured using an electronic spirometer (pocket micro-spirometer, VIASYS Healthcare GmbH, D-97,204 Hoechberg, Germany) [20]. The subject was asked to inhale as much air as possible and then exhale forcefully and as quickly as possible into the mouthpiece of the spirometer. The test was repeated three times. The best of the three reproducible test results was recorded and compared with Sudanese reference spirometric values [21]. The aim of this study was to detect changes in pulmonary function testing in different drug users.

#### Data Analysis

The data were analyzed using SPSS software, version 25. A one-sample t-test was used for comparison with reference values for pulmonary function tests. Correlations between variables were estimated by the coefficient of determination (r) using the bivariate Pearson correlation coefficient. The Kruskal-Wallis test and the Wilcoxon signed-rank test were used for analysis when comparing the results between the spirometric values of the abusers and their matched normal Sudanese, depending on the non-normal distribution of the data, and a *P* value ≤ 0.05 was considered statistically significant.

## Results

### Demographic data

Sixty substance users were included in this study: 55 males and 5 females. The mean age was 31 ± 5.5 years, and the mean height was 176 ± 5.5 cm. (Table 1)

**Table 1:** Age, height, duration and type of substance use among substance users (*n* = 60):

Variable	Mean ± SD	Minimum	Maximum
**Age/years**	**31 ± 5.5 years**	**18 years**	**55 years**
**Height /cm**	**176 ± 5.5 cm**	**150 cm**	**195 cm**
**Duration of uses/ months**	**54 ± 6.3months**	**1 month**	**30 years**
**Type of substance**	**Frequency**	**Percent**	**Mean ± SD years**
**Alcohol**	**12**	**20%**	**42 ± 5.5**
**Cannabis**	**16**	**26.6%**	**26 ± 5.5**
**Heroin**	**16**	**26.6%**	**31 ± 5.5**
**Methamphetamine**	**16**	**26.6%**	**26 ± 5.5**
**Total**	**60**	**100%**	**31 ± 5.5**

We divided the sample into different age groups: the first group between 18 and 30 years (*n* = 29), the second group between 31 and 40 years (*n* = 23), and the third group between 31 and 55 years (*n* = 8).

43 participants had a free medical background. There were nine participants with hypertension, four with diabetes, two with thyroid disease, and two with heart disease. (All participants are under regular treatment.)

### Addiction and substance uses data

This study included four groups: cannabis users (*n* = 16), heroin users (*n* = 16), methamphetamine users (*n* = 16), and alcohol users (*n* = 12). The mean duration of substance use was 54 ± 6.3 months for all groups. (Table 1)

Of the users, 31 were active cigarette smokers, 10 were water pipe (shisha) smokers, and 19 were non-smokers.

**Fig. 1:**
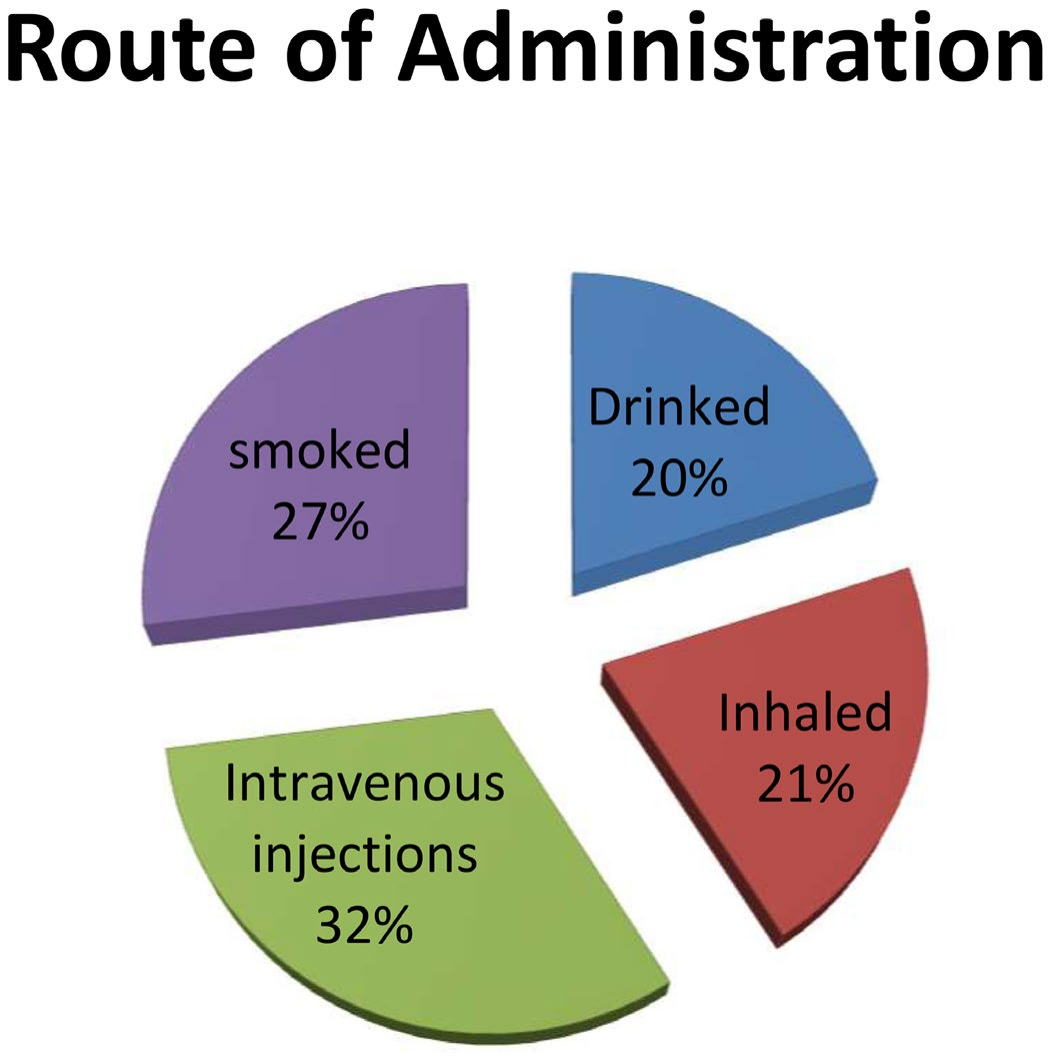
Show the prevalence of different route of administrations used in substance use (*n* = 60)

53% of participants used the intravenous or inhaled route of drug abuse. (Fig. 1)

### PFTs among the substance abusers

**Fig. 2:**
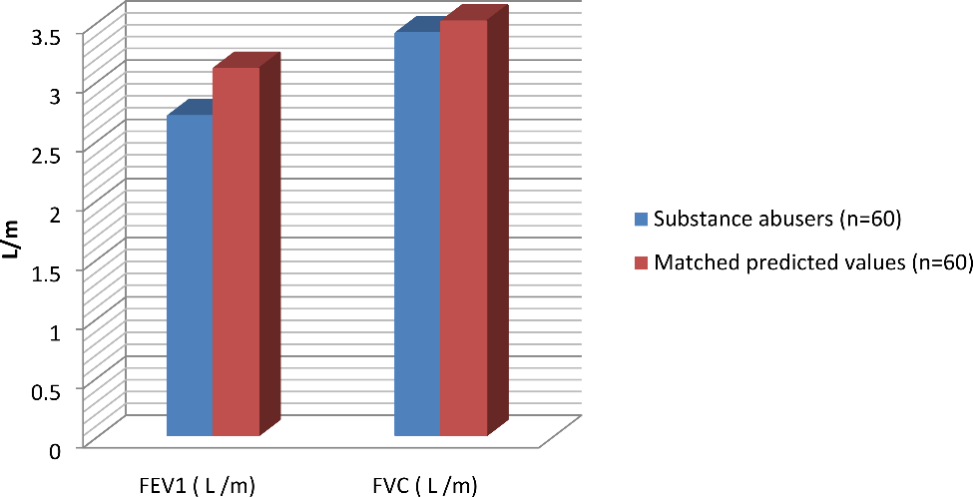
FEV1 and FVC in substance abusers and their matched predicted values (control) [21]

All PFT parameters were found to be lower in substance abusers compared to their matched controls, and only the FEV1/FVC ratio was significantly affected. (Table 2; Fig. 2)

**Fig. 3:**
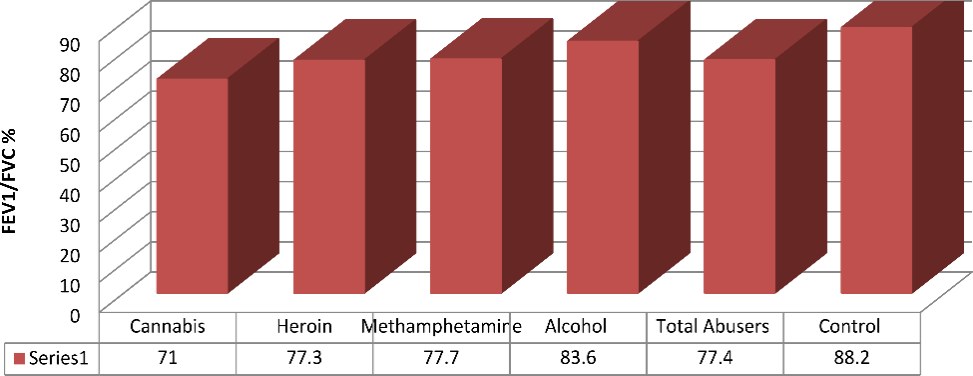
Mean of Spirometric measures across different substance abuse groups compared to normal spirometric values (control) [21]

The lowest FEV1/FVC ratio was found in cannabis users (mean 71.03 ± 11.3%) (P 0.001) (Table 2). Significant differences in FEV1/FVC ratio were found between different substances of use and controls (P 0.001) for cannabis, (P 0.008) for heroin, (P 0.013) for methamphetamine, and (P 0.013) for alcohol. 81.6% of users had an FEV1/FVC ratio of less than 70%. (Fig. 3)

**Table 2:** Comparison of The PFTs parameters between the substance abusers and their matched predicted values

Parameter (Mean ± SD)	Substance abusers (*n* = 60)	Matched predicted values (*n* = 60)	*P*- Value
**FEV1 (L /m)**	**2.7 ± 0.64**	**3.1 ± 0.27**	**0.221**
**FVC (L/m)**	**3.4 ± 0.40**	**3.5 ± 0.28**	**0.93**
**FEV1/ FVC (%)**	**77.4 ± 13.1**	**88.2 ± 2.9**	**0.001**
**PEFR (ml/sec)**	**449.8 ± 66.5**	**450.1 ± 49.4**	**0.974**

*Wilcoxon Signed Rank test was used to compare values from both groups

There was also a significant difference in FEV1/FVC ratio between routes of administration between inhaled (77.1 ± 16.9) and smoked (71 ± 11.3) (P 0.01), intravenous injection (77.9 ± 16.7) and oral (83.6 ± 7.6) (P 0.001). (Table 2)

**Table 3:** Mean Distribution of Spirometric measures across different substance abuse groups

Type of substance used	***N***	FEV1 Mean ± SD	FVC Mean ± SD	PEFR Mean ± SD	FEV1/FVC Mean ± SD
**Alcohol**	**12/60**	**2.9 ± 0.37**	**3.5 ± 0.18**	**449.7 ± 24.8**	**83.6 ± 7.6**
**Cannabis**	**16/60**	**2.6 ± 0.59**	**3.4 ± 0.40**	**451.3 ± 69.8**	**71.03 ± 11.3**
**Heroin**	**16/60**	**2.6 ± 0.86**	**3.3 ± 0.64**	**450 ± 100.7**	**77.3 ± 15.8**
**Methamphetamine**	**16/60**	**2.7 ± 0.77**	**3.4 ± 0.38**	**448.5 ± 70.9**	**77.7 ± 17.8**
***P*** **value (Kruskal walis test)**		**0.221**	**0.932**	**0.974**	**0.001**
**Route of administration**	***N***	**FEV1** **Mean ± SD**	**FVC** **Mean ± SD**	**PEFR** **Mean ± SD**	**FEV1/FVC** **Mean ± SD**
**Orally**	**12/60**	**2.9 ± 0.37**	**3.5 ± 0.18**	**449.7 ± 24.8**	**83.6 ± 7.6**
**Inhaled**	**13/60**	**2.4 ± 0.86**	**3.1 ± 0.54**	**447.3 ± 117.8**	**77.1 ± 16.9**
**Intravenous injection**	**19/60**	**2.9 ± 0.74**	**3.6 ± 0.39**	**450.9 ± 50.8**	**77.9 ± 16.7**
**Smoked**	**16/60**	**2.6 ± 0.59**	**3.4 ± 0.49**	**448.3 ± 69.8**	**71 ± 11.3**
***P*** **value (Kruskal walis test)**		**0.068**	**0.107**	**0.932**	**0.001**
**Age groups**	***N***	**FEV1** **Mean ± SD**	**FVC** **Mean ± SD**	**PEFR** **Mean ± SD**	**FEV1/FVC** **Mean ± SD**
**18–30 years**	**29/60**	**2.9 ± 0.23**	**3.3 ± 0.65**	**448 ± 2.3**	**82.8 ± 2.19**
**31–40 years**	**23/60**	**2.7 ± 1.46**	**3.6 ± 0.37**	**450 ± 13.6**	**75 ± 1.4**
**41–55 years**	**8/60**	**2.5 ± 0.36**	**3.1 ± 0.62**	**447 ± 12**	**80.6 ± 4.5**
***P*** **value (Kruskal walis test)**		**0.04**	**0.14**	**0.174**	**< 0.001**
**Chronic diseases**	***N***	**FEV1** **Mean ± SD**	**FVC** **Mean ± SD**	**PEFR** **Mean ± SD**	**FEV1/FVC** **Mean ± SD**
**Heart diseases**	**2/60**	**2.05 ± 0.07**	**3.5 ± 0.07**	**448 ± 21.2**	**57.6 ± 0.91**
**HTN**	**9/60**	**2.7 ± 0.79**	**3.5 ± 0.63**	**447.7 ± 31.3**	**77.3 ± 13.12**
**DM**	**4/60**	**2.5 ± 1.04**	**3.17 ± 0.43**	**449 ± 80.6**	**69.3 ± 14**
**Thyroid diseases**	**2/60**	**3.1 ± 0.21**	**3.6 ± 0.14**	**450 ± 14.1**	**86.4 ± 2.19**
**None (Free from medical disorders)**	**43/60**	**2.7 ± 0.65**	**3.4 ± 0.4**	**450.1 ± 81.3**	**77.7 ± 14.48**
***P*** **value (Kruskal walis test)**		**0.057**	**0.406**	**0.146**	**< 0.001**
**Social habits**	***N***	**FEV1** **Mean ± SD**	**FVC** **Mean ± SD**	**PEFR** **Mean ± SD**	**FEV1/FVC** **Mean ± SD**
**Cigarette**	**31/60**	**2.8 ± 0.72**	**3.4 ± 0.55**	**447.03 ± 80.2**	**77.8 ± 13.7**
**Shisha**	**10/60**	**2.5 ± 0.78**	**3.4 ± 0.32**	**448.6 ± 78.9**	**72.3 ± 18.9**
**None Smokers**	**19/60**	**2.7 ± 0.58**	**3.4 ± 0.34**	**450.5 ± 56.3**	**78.17 ± 12.7**
***P*** **value (Kruskal walis test)**		**0.090**	**0.997**	**0.091**	**< 0.001**

### Effects of age, substance duration and chronic diseases in the PFTs among substance abusers

FEV1 was found to be significantly affected by age (P 0.004). 80.0% of abusers had an FEV1/FVC ratio of less than 75%, with the most common age group being 18–30 years (Table 3).

**Table 4:** Show the effect of substance use on FEV1/FVC across different categories (*N* = 60):

Age group	Number	FEV1/FVC < 75% *N* (%)	FEV1/FVC ≥ 75% *N* (%)
**18–30 years**	**29**	**25 (86.2%)**	**4 (13.7%)**
**31–40 years**	**23**	**18 (78.2%)**	**5 (21.7%)**
**41–55 years**	**8**	**5 (62.5%)**	**3 (37.5%)**
**Total**	**60**	**48 (80%)**	**12 (20%)**
**Substance uses**	**Number**	**FEV1/FVC < 75%** ***N*** **(%)**	**FEV1/FVC ≥ 75%** ***N*** **(%)**
**Cannabis**	**16**	**15(93.7%)**	**1(6.25%)**
**Heroin**	**16**	**9(56.2%)**	**7(43.7%)**
**Methamphetamine**	**16**	**14(87.5%)**	**2(12.5%)**
**Alcohol**	**12**	**10(83.3%)**	**2(16.6%)**
**Total**	**60**	**48(80%)**	**12(20%)**
**Social habits**	**Number**	**FEV1/FVC < 75%** ***N*** **(%)**	**FEV1/FVC ≥ 75%** ***N*** **(%)**
**Cigarette**	**31**	**26 (83.8%)**	**5(16.1%)**
**Water pipe (Shisha)**	**10**	**9(90%)**	**1(10%)**
**Total (Cigarette + Shisha)**	**41**	**35(85.3%)**	**6(14.6%)**
**None smokers**	**19**	**13(68.4%)**	**6(31.5%)**
**Total Abusers**	**60**	**48(80%)**	**12(20%)**

The duration of substance use was inversely correlated with the FEV1/FVC ratio (r=-0.378, P 0.003), suggesting that a longer duration of substance use is associated with lower FEV1/FVC ratios.

There was a significant difference between categories with chronic diseases (only 17 patients) about the FEV1/FVC ratio, with the lowest values for those with heart disease (57.6 ± 0.91) (P 0.001) (Table 2).

To investigate whether the reduction in FEV1/FVC ratio among substance abusers is related to the confounding effect of chronic diseases or not, we used Wilcoxon signed rank (Mann-Whitney U test) to see the differences in FEV1/FVC ratio among medically free abusers (43 participants) and their matched control, and a significant *p*-value (0.004) was reported, indicating the effects of substance abuse on PFTs.

### Effects of smoking on the PFTs among substance abusers

Shisha users reported the lowest FEV1/FVC ratio (72.3 ± 18.9%) with a significant p-value of (< 0.001), indicating that shisha use in addition to substance abuse has a worsening effect on PFTs, (Table 2).

85.3% of the smokers (cigarettes, shisha, and substance abuse) reported a FEV1/FVC ratio less than 75%, and 14.6% of them reported a FEV1/FVC ratio greater than 75%. On the other hand, 68.4% of non-smokers reported a FEV1/FVC ratio of less than 75%, suggesting that the effect of substance abuse on the FEV1/FVC ratio is greater than that of smoking.

To investigate whether the reduction in FEV1/FVC ratio among substance abusers is related to the confounding effect of smoking or not, we used Wilcoxon signed rank (Mann-Whitney U test) to see the differences in FEV1/FVC ratio among non-smoking abusers and their matched controls, and a significant p-value (0.002) was reported indicating the effect of substance abuse on PFTs. In addition to that, 68.4% of non-smoking abusers reported an FEV/FVC ratio less than 75%, (Table 3).

### Pattern of respiratory defects among substance abusers

**Fig. 4:**
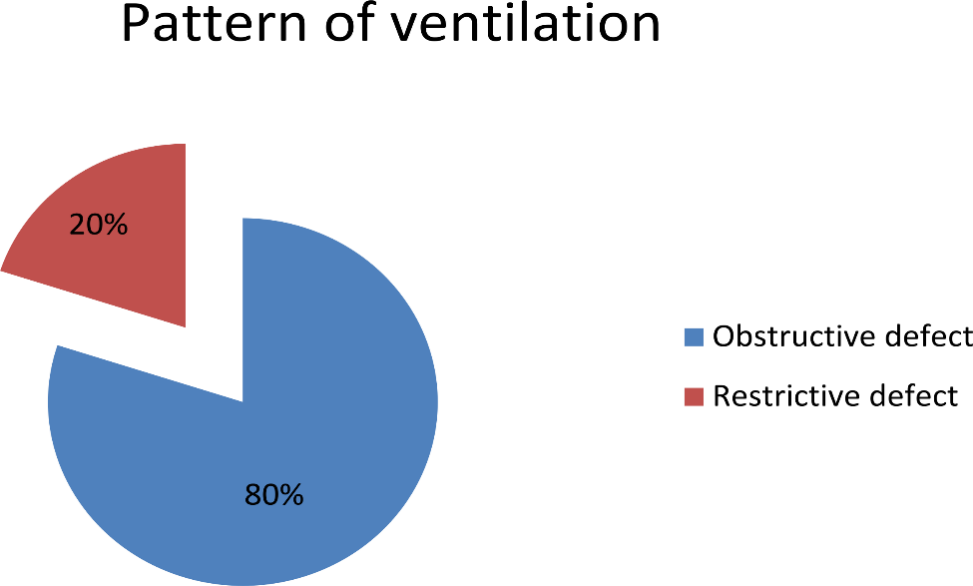
Pattern of ventilation among substance abusers (*n* = 60)

No participant had a normal result in all PFT parameters, and all sixty abusers (100.0%) had abnormal PFTs. 48 of the abusers (80.0%) recorded FEV1/FVC < 75%, indicating an obstructive lung defect. Twelve of the abusers (20.0%) recorded FEV1/FVC ≥ 75%, indicating restrictive defect abnormalities, as shown in Fig. 4.

## Discussion

In recognition of the perceived increase in substance use in Sudan and the lack of knowledge about the health problems affecting this population. Most previous studies conducted in Sudan have focused mainly on cigarette smoking. To the best of our knowledge, none have been conducted on different substance users. This study determines the effects of different types of substance use on lung function in Abdalaal Elidrisi Psychiatric Hospital. All FEV1/FVC ratios studied in our different groups were low compared with published reference values; we compared the results with published Sudanese reference normal values of the same age and height.

Sixty drug users were included in this study. The average age is 31 ± 5.5 years. Although drug use has reached even younger members of society, which will increase the negative effects of drug use in many ways.

The number of female drug users has increased significantly. However, few of them seek medical advice and visit rehabilitation centers, which could be explained by the reluctance of families due to fear of stigma and social opinion.

Cannabis has the lowest FEV1/FVC ratio compared to all substance groups. Our results were in agreement with the previous study by Aldington et al., who found that cannabis users had evidence of airflow obstruction as measured by the FEV1/FVC ratio [9]. This result differed from a study by Tashkin et al., which showed that there was no correlation between the decrease in FEV1/FVC ratio and cannabis use [22]. However, an earlier study by Bloom et al. showed increased FEV1 and FVC in cannabis smokers compared to non-smokers [23]. In the present study, there was a significant reduction in the FEV1/FVC ratio of the cannabis group compared with their age- and height-matched reference values.

Regarding the PEFR in cannabis users, the results in our study were within the normal range compared to healthy controls. In a study of 350 subjects, the PEFR was higher in cannabis users than in controls, possibly because short-term cannabis use is associated with bronchodilation [23]. Cannabis exposure has an immediate and modest bronchodilator effect, with a subsequent increase in airway inflammation and symptoms of chronic bronchitis. The acute bronchodilator effect appears to be due to tetrahydrocannabinol (THC), the psychoactive ingredient in marijuana [25].

Studies investigating the effect of smoking marijuana on lung function have produced somewhat mixed results [2, 10, 25]. This is likely to be related to the difficulty in obtaining samples of smokers due to their illegal status. In addition, correction for variables such as tobacco smoking may be difficult or virtually impossible in the case of differences in smoking technique.

Our heroin users had a reduced FEV1/FVC ratio. Furthermore, increasing the duration of use led to a greater reduction. This significant decrease in FEV1/FVC may indicate a tendency towards an obstructive pattern, despite the exclusion of all known cases of obstructive lung disease.

Consistently, Burhan et al. reported similar detrimental effects of heroin use on function by increasing the risk of developing obstructive airway disease [26]. However, another study suggested that heroin users have a high and increasing burden of chronic respiratory symptoms and a decline in FEV1 that exceeds the normal age-related decline observed in healthy non-users [8].

With the increasing number of methamphetamine users in Sudan, the responsibility of researchers and medical teams to know the health dimensions of this dangerous substance in order to make appropriate medical decisions increases. In our study, methamphetamine users had decreased FEV1/FVC, which makes further research necessary and raises new questions about the mechanisms by which methamphetamine affects health. There wasn’t enough data on the effects of methamphetamine use on function. However, one study suggested that there was no correlation between FEV1, FVC, FEV1/FVC ratio, and PEFR in methamphetamine users and non-users and a weak correlation between duration of methamphetamine use and a decrease in FEV1/FVC ratio [4].

In our alcoholics, there was a significant difference in the FEV1/FVC ratio between alcohol users and normal, healthy controls, with lower ratios in alcohol users compared with healthy controls. Rankin et al. studied 70 alcoholics, found airway obstruction in half of them, and concluded that it was secondary to cigarette smoking. However, many studies have been done on alcoholics, but most of the subjects were also tobacco smokers, with varying amounts and durations of use [27].

### Limitations of the study

This was a cross-sectional study, and participants were not followed up. Baseline data on drug users in Sudan was not available. Urine for drug toxicology is the only available test, so the amount of substance at the time of PFT is unknown. A retrospective cohort study (to avoid ethical issues) is recommended to see the effects of different drug abuse concentrations on PFTs. The lack of female participants and the fact that drug abuse is not socially acceptable for women in traditional Sudanese society contributed to the study’s social challenges. Few of them seek medical counsel or go to treatment facilities, which may be attributed to families’ hesitation out of concern for societal stigma and opinion. Instead, most families choose to treat their female addicts at home.

## Conclusions

Different types of substances (cannabis, methamphetamine, heroin, and alcohol) have harmful effects on the pulmonary system, as indicated by a reduction in the measured FEV1/FVC ratio. This puts the individual at risk of developing obstructive airway disease. Among cannabis smokers, those with a longer duration of use and those with social habits (shisha) were at greater risk. Substance abusers may have different patterns of lung function impairment, with an obstructive pattern being the most common. This should be a top public health priority and indicates an urgent need for increased awareness.

## Data Availability

The data generated in this study are available from the corresponding author upon reasonable request with a completed Materials Transfer Agreement, excluding the materials including personally identifiable information.
